# Opportunities and Challenges in Flexible and Stretchable Electronics: A Panel Discussion at ISFSE2016

**DOI:** 10.3390/mi8040129

**Published:** 2017-04-18

**Authors:** Zhigang Wu, Yongan Huang, Rong Chen

**Affiliations:** 1 State Key Laboratory of Digital Manufacturing Equipment and Technology, Huazhong University of Science and Technology, Wuhan 430074, China; yahuang@hust.edu.cn (Y.H.); rongchen@mail.hust.edu.cn (R.C.); 2 Flexible Electronics Research Center, Huazhong University of Science and Technology, Wuhan 430074, China

The 2016 International Symposium of Flexible and Stretchable Electronics (ISFSE2016), co-sponsored by the Flexible Electronics Research Center, Huazhong University of Science and Technology (HUST) & State Key Laboratory of Digital Manufacturing and Equipment Technology, National Natural Science and Engineering (NSFC), was successfully held in Wuhan, China, 29–30 June 2016.

A panel of five scholars of international standing led the panel discussion at the conference on important and timely topics including reliability or new functions of flexible and stretchable electronics, advanced materials, flexible electronics manufacturing, ways to link flexible electronics to medical applications, and the roles of organic and inorganic electronics in flexible electronics.


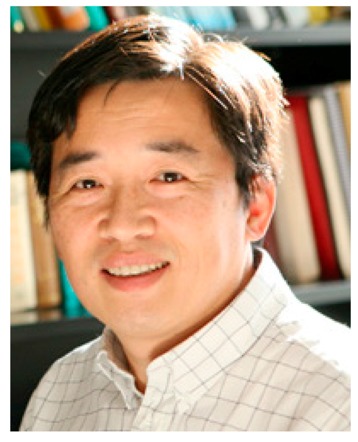
Dr. Yonggang Huang is the Walter P. Murphy Professor of Mechanical Engineering, Civil and Environmental Engineering, and Materials Science and Engineering at Northwestern University. He is interested in the mechanics of stretchable and flexible electronics, and 3D fabrication of complex materials and structures. He is a member of the National Academy of Engineering, USA.
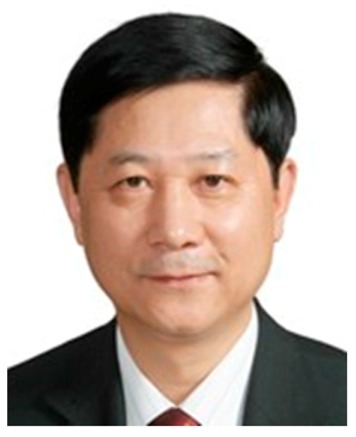
Dr. Yibing Cheng is a professor of Materials Science and Engineering at Monash University. He specializes in inorganic materials. He has particular interests in the development of solution processed solar cells, especially by printing. He is a fellow of the Australian Academy of Technological Sciences and Engineering.
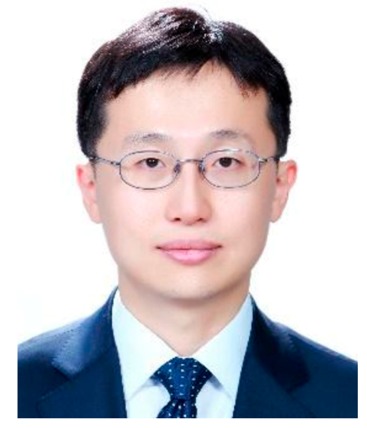
Dr. Dae-Hyeong Kim is an associate professor of chemical and biological engineering at Seoul National University. His research aims to develop technologies for high-performance flexible and stretchable electronic devices using high-quality single crystal inorganic materials and novel biocompatible materials that enable a new generation of implantable biomedical systems with novel capabilities and increased performance.
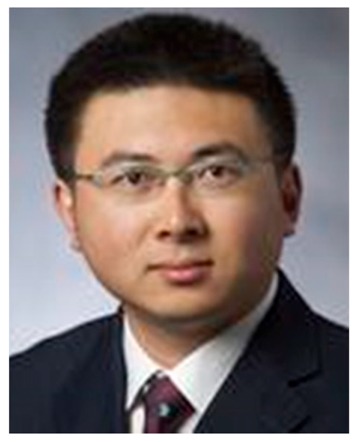
Dr. Xuanhe Zhao is an associate professor and Noyce Career Development Chair in the Department of Mechanical Engineering, MIT. His current research goal is to understand and design soft materials that possess unprecedented properties and functions. Dr. Zhao is a recipient of the NSF CAREER Award, the ONR Young Investigator Award, and the Early Career Researchers Award from AVS Biomaterial Interfaces Division.
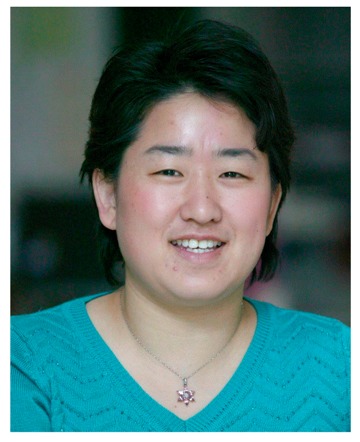
Dr. Haixia Zhang is a professor in the Institute of Microelectronics, Peking University. Her research fields include micro/nano energy harvesting technologies, self-powering systems and active sensors. She has published more than 100 papers in prominent journals, six books/book chapters and 30 patents on micro/nanotechnology.

1. What are the key challenges in flexible electronics? What is the niche market and the killer application for stretchable electronics?

Huang: One killer application of flexible or stretchable electronics is in medicine. Dae-Hyeong, you have done a lot of work in this area, can you make some comments?

Kim: I totally agree with Prof. Huang. The limitation of flexible and stretchable electronics is that the device performance may be lower than conventional rigid electronics. Flexible and stretchable electronics may not be able to compete with rigid electronics in device performances. When we change the substrate from the rigid silicon wafer to plastics, the device performance would be decreased significantly. Therefore, what we need to do is to find out new markets and applications, such as novel medical systems based on flexible electronics. In medical applications, the device should be human-friendly. Individual organs and/or tissues are quite soft, and their shape is curvilinear. So, the device should also be deformable to conform to these biological systems, which is a key property of flexible and stretchable electronics.

Zhao: I fully agree with Yonggang and Dae-Hyeong’s points. In the health care industry, I think flexible and stretchable electronics indeed has a niche market. I also want to add some additional points. Flexible and stretchable electronics may address some critical issues in this aging society, in addition to health care; for example, monitoring the well-being of senior people. We will probably not pay a thousand dollars or more to buy a flexible cell phone. But if the cell phone were able to be used as a very comfortable device for senior people and were able to monitor many of their vital signals, we might buy it. Another potential application is in education. We now learn many new things through cell phones. But it would be better if we could have a more conformal and natural way to receive different types of information.

Huang: I totally agree with your points on health care. But I do not really follow your comment on education. I am not sure I understand it yet.

Zhao: Books and rigid devices such as tablet computers have allowed us to learn new information. Flexible electronics, for example, flexible goggles, and different types of virtual reality devices that are more flexible and more conformable, may even do better in this regard. Learning is no longer limited to the classroom; learning is everywhere. I think this kind of conformal devices that can be very comfortably attached to your body may lead to innovative ways of learning and education.

Huang: I fully agree with what you have just said.

Zhang: I want to add some comments here again. So, regarding health care, I agree, we should certainly pay more attention to the wearable market. Another hot topic is artificial intelligence (AI). Recently, AI has become very popular everywhere—such as in famous human-like robots that resemble the most beautiful ladies—and draws a lot of attention. So, if we see this as the future of AI, the future of robots, then soft electronics and conformal electronics are exactly what is needed. If we can put all these on top of our skin, they can detect not only temperature but also many other parameters. Then, these robots will be much smaller in many conditions. I think we should pay attention to these advanced technologies.

2. Is polydimethylsiloxane (PDMS) the best carrier? Or hydrogel? What is the area of greatest potential for hydrogel?

Huang: I do not think there is one best carrier for everything. There are so many substrates that we have used, such as PDMS, Ecoflex**^®^**, and Silbione**^®^**, depending on the applications. I do not think we need to identify one single material that suits all the purposes. For example, the elastic modulus of Ecoflex matches well with that of the skin, and is therefore useful for epidermal applications. For some other applications, PDMS may be a good choice.

Zhao: I agree with Yonggang. The choice of material is application-dependent, especially when considering integrating devices with the human body. Different parts of human bodies have different properties, so this is a material design and system design issue. PDMS has been widely used in flexible electronics, while hydrogels have broad applications in biomedicine. But I do not think there is a best material; it will depend on the application.

Huang: Dae-Hyeong uses one special type of substrate, silk, for bio-integrated electronics, which can dissolve inside the human body. This cannot be achieved by PDMS. Therefore, different applications require different substrates.

3. Shall we focus more on the reliability or new functions?

Cheng: I think it very much depends on what your interest is. If you are interested in applications, I think the reliability is probably more important for consideration. Many excellent research works about different flexible or stretchable electronics have already been reported, but most of these works have not been accepted by the market as commercial products, because most of them failed in reliability or in functionality. For example, I just talked about perovskite solar cells, showing 20% efficiency. But their long-term performance is poor, currently lasting for two weeks or a month. So, from that point of view, I think it would be a huge contribution to the whole field if we can improve the reliability of one or two research outcomes and make them really accepted by the market. However, if you are a young researcher, just coming into this field, and you want to do something exciting or achieve good publications, then focusing on reliability may not be your best choice. This is because the study of reliability is time-consuming and may be difficult to produce many journal papers with very high impact factor. So, if you really want to publish in Nature or Science, you would probably prefer to work on something that is new and more exciting.

Zhang: From my experience in my field, MEMS (Micro-Electro-Mechanical System) is probably not a good field for publication. In most MEMS journals, the impact factor is pretty low, for example, below 2. So, that presents many issues for students, because they want to graduate with very good records. Especially in China, a lot of university academics now asking for high impact publications. So, if you are a student, I strongly suggest that you work on the functions and innovations, and offer some new innovations for good publications. That is not only to make your resume look impressive, it is also very good educational training. At a very young age, you should find something really exciting, not invest your time in something reliable. Reliability research is not for the young students in the lab. So I suggest, if you are working in the lab, and still want to continue academia career as a professor, you should try your best to make something new, try to make something really exciting. After many years, you may move into a big production market, and then you will be able to hire a bunch of people to work on the reliability. I think that is the strategy.

Zhao: For a wide range of applications of flexible and stretchable electronics, reliability is a very import issue. The first light bulb invented by Thomas Edison only lasted a few hours, which probably would not be widely adopted in the society. Edison and his team further improved the design so that it could last over a thousand hours, and then the widespread applications of light bulks made a major impact on our society. For the field of flexible and stretchable electronics, we do need to invent new functions and applications. At the same time, especially for translational and industrial applications, we also need to pay attention to the reliability.

Kim: For students, I think that innovation should be emphasized. It is true that reliability is always important. For example, all the medical devices that we are working on always need to be highly reliable. But I personally think that new ideas and innovations are more important, as we are conducting research at the university. Of course, if you are working at a company, reliability is very important. However, we are working on new ideas, and creating new frontiers and new innovations. So, students should be working more on new, innovative functions.

4. Is it of interest to actuating technology? For what kind of applications?

Kim: Ok, usually I work on soft medical devices, and it is related to sensors and actuators. For the sensing purpose, sometimes we need actuators. If you just want to measure the electrical features, an amplifier might be good enough. But if you want to measure some mechanical properties of specific tissues or organs, then appropriate actuation gives us better sensing results. We need to combine sensors and actuators for a better quality diagnosis. Meanwhile, I think that in the future soft robotics will be a hot field. In the past, robots were rigid. But in the future, we imagine soft, human-like robots. And in that case, we will probably need soft actuators.

Zhang: Actually, it is very important for smart systems. Now, it is very popular to design mini robots to put inside the body, or inject into the body to make detections and perform some surgeries. For that purpose, robots should have internal actuators. I have not worked on these actuators, but I do think they are a very important field and research direction.

Kim: I have something to add. Actuators are important in medical systems. For example, the brain and heart are organs operated by electrical signals. And electrical impulses and stimulations can be used to treat many diseases related to the brain and heart. Also, drug delivery should be controlled by appropriate actuators for controlled drug release.

5. What are the unsolved problems in flexible electronics manufacturing?

Huang: Flexible electronics manufacturing is in its infancy, and there are numerous unsolved problems in this area. In fact, the United States government investigated $70M to form a center on the manufacturing of flexible electronics.

Zhao: Manufacturing of flexible electronics requires different techniques. Is 3D printing a possible technique for the fabrication of flexible and stretchable electronics?

Huang: I will answer this. 3D printing can print polymers and some metals, but it can never print single crystal silicon or other important materials for electronics.

Zhao: How about 3D printing plus transferring?

Huang: This is an interesting idea. Would you like to explain a bit more?

Zhang: 3D printing is good but it is not suitable for mass production. So, if we want to make electronics that are flexible, we must use this kind of traditional technology and basic tools. Therefore, I agree with Yonggang’s comments; it is important that fabrication technology tries to use all these existing successful technologies and makes something based on them. This will be easy for mass production and for the actual industry.

Huang: It is important to take advantage of the existing, mature semiconductor fab to develop inorganic, flexible and stretchable electronics.

Cheng: I think printing technologies could be used for flexible electronics manufacturing. 3D printing has potential in the flexible electronics field, such as for health-related products. Many health-related flexible products are associated with individual people. So, for these kinds of products, 3D printing is probably quite suitable. This is a quite new field and we do not need to restrict our minds and initiatives. I guess the point is that while recognizing the existing technologies, such as silicon technology, we should not be afraid of trying and integrating new things.

6. How can we link flexible electronics to medical applications?

Kim: I think that, at least, we need to develop a completely new device that can solve critical issues which conventional devices cannot solve.

Huang: Or you can make medical electronics flexible so that they can be applied at home.

Kim: Yes, I think that such applications are good candidate markets. We need to persuade medical doctors and patients to use our devices. To do that, the performance of the flexible medical device should be much better than existing commercial technologies. In the medical area, there are many diseases that cannot be cured with existing technologies. In that case, we can create new devices with unconventional functions to address those unmet needs.

Huang: Thank you. In this development, it is really important to work with medical doctors.

Zhang: I think something must be pointed out. My thought is that flexible electronics has great potential for Chinese medicine. Normally, diagnoses are taken by very old doctors. They always follow four ways of diagnosis: look, listen, question and feel the pulse, which are based on their experiences. So, if we can put flexible electronics on the body, if we can monitor these parameters, then a lot of data could be recorded and integrated with these old doctors’ experiences. I think that can solve a big problem for Chinese medicine. I think for young students, if they have the chance, they should focus more on these applications. They have a really big future.

Kim: I want to add one point. To translate flexible devices to medical applications, we need to publish articles in medical journals. Although these devices are for general people, the person who needs to decide whether that device can be applied to patients is a medical doctor. And through medical journals, we can let medical doctors know the advantages of our new medical device technology. 

Zhao: I want to add another point, regarding the patent. The protection of intellectual property is extremely important in this field. Once a technology is mature enough, we need to consider patenting it. So, when doctors and companies approach you, you or your team will have the intellectual property to proceed with the commercialization of the technology.

7. What are the roles of organic electronics or inorganic electronics in the field of flexible electronics?

Cheng: In the past, many functional devices were made of inorganic electronics. But in the last ten or fifteen years, there has been an increasing amount of new polymers and new organic materials synthesized and applied to functional electronics. I think there is a catch here. If you favor flexible devices, then you will consider your substrate to be normally of polymer or organic material. If you want to work on organic substrates, then in many cases, it is often a challenge to make devices on organic substrates and some new manufacturing techniques must be developed. For example, you cannot apply high temperature with polymer substrates, which in some cases restricted the application of inorganic materials. Both organic and inorganic materials are important, depending on their functions. But in many cases, they are restricted by processing technology. You may wish to use inorganic materials for flexible devices, however you may not have a suitable processing technology. If new technologies, such as the femto-second laser, can be used, then you can avoid high temperature heating, which can be applied to many organic substrates. As a result, many previously unthinkable inorganic materials may be applicable to polymer substrates.

Huang: Organic and inorganic materials both have their niche applications. Flexible display is an excellent example of organic electronics because one’s eyes cannot react in less than 0.1 second anyway, such that the relatively low charge mobility for organics would not be an issue. It is important to develop both organic and inorganic flexible electronics.

8. Is it possible to utilize the roll to roll technology in stretchable electronics? How?

Cheng: I think roll to roll is an ideal, fast and low-cost technology. However, roll to roll is very much restricted by the substrates. For example, we cannot use roll to roll to print on glass. Of course, on flexible glass maybe, but it is very fragile. Now, if you work on stretchable devices, then that is a great challenge because stretchable means that during the roll to roll processing, materials may be deformed, which would cause enormous problems in roll to roll processing.

Zhang: Just as Prof. Cheng said, this really depends on the material. Another point for roll to roll, is how to make multi-layers. If we make several layers, then roll to roll has a big issue with mismatch; you could not achieve very good results. So, I think it depends on the device and structural design.

Cheng: What is the purpose of roll to roll technology? Roll to roll can be fast, but today, it is not the only technology to achieve fast production and low-cost. So, I don’t think that roll to roll technology is an objective; it is only a process. You have many other choices as well. For example, I think that stretchable electronics may not need roll to roll technology in order to make the device cheaper. It is not necessary.

